# Exome-First Approach in Fetal Akinesia Reveals Chromosome 1p36 Deletion Syndrome

**DOI:** 10.1155/2019/6753184

**Published:** 2019-10-02

**Authors:** Masatake Toshimitsu, Shinichi Nagaoka, Shuusaku Kobori, Maki Ogawa, Fumihiko Suzuki, Takema Kato, Shunsuke Miyai, Rie Kawamura, Hidehito Inagaki, Hiroki Kurahashi, Jun Murotsuki

**Affiliations:** ^1^Department of Maternal and Fetal Medicine, Miyagi Children's Hospital, Miyagi, Japan; ^2^Division of Molecular Genetics, Institute for Comprehensive Medical Science, Fujita Health University, Toyoake, Japan

## Abstract

**Background:**

Fetal akinesia refers to a broad spectrum of disorders with reduced or absent fetal movements. There is no established approach for prenatal diagnosis of the cause of fetal akinesia. Chromosome 1p36 deletion syndrome is the most common subtelomeric terminal deletion syndrome, recognized postnatally from typical craniofacial features. However, the influence of chromosome 1p36 deletion on fetal movements remains unknown.

**Case Report:**

A 32-week-old fetus with akinesia showed multiple abnormalities, including fetal growth restriction, congenital cardiac defects, and ventriculomegaly. G-banding analysis using cultured amniocytes revealed 46,XY,22pstk+. Postnatal whole exome sequencing and subsequent chromosomal microarray identified a 3 Mb deletion of chromosomal region 1p36.33–p36.32. These results of molecular cytogenetic analyses were consistent with the fetal sonographic findings.

**Conclusion:**

Using the exome-first approach, we identified a case with fetal akinesia associated with chromosome 1p36 deletion. Chromosome 1p36 deletion syndrome may be considered for differential diagnosis in cases of fetal akinesia with multiple abnormalities.

## 1. Introduction

Fetal akinesia is a condition characterized by reduced or absent fetal movements independent of the etiologies [1–3]. A definitive diagnosis of the cause could be helpful for perinatal management, perinatal decision-making within local limits, and genetic counseling for future pregnancies [1]. Although next-generation sequencing (NGS) technologies have identified some underlying genetic mutations associated with fetal akinesia, some cases remain genetically unsolved [2, 3].

Chromosome 1p36 deletion syndrome (MIM# 607872) is the most common subtelomeric terminal deletion syndrome with a prevalence of 1 : 5000 newborns [4]. The typical clinical features of this syndrome include generalized hypotonia, severe developmental delay, seizure, growth restriction, microcephaly, congenital heart defects, flat nasal bridge, and midface hypoplasia [4]. It is classically diagnosed postnatally from typical craniofacial features, although prenatal characteristic findings have been described [4, 5]. The recurrence risk depends on the mechanism of the deletion, such as *de novo* deletion or inheritance from one of the parents with balanced translocations [6].

Here, we present a case with fetal akinesia associated with chromosome 1p36 deletion syndrome, which was not suspected from prenatal clinical findings before genetic testing and was diagnosed postnatally by the exome-first approach.

## 2. Clinical Case

**Figure 1:**
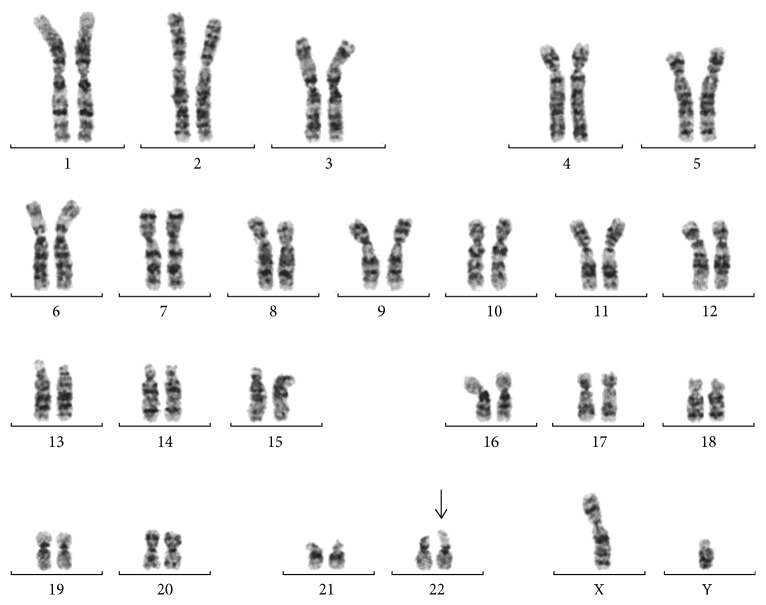
G-banding analysis of cultured amniocytes at 32 weeks and 6 days of gestation. The fetal karyotype was 46,XY,22pstk+. The arrow indicates 22pstk+.

**Figure 2:**
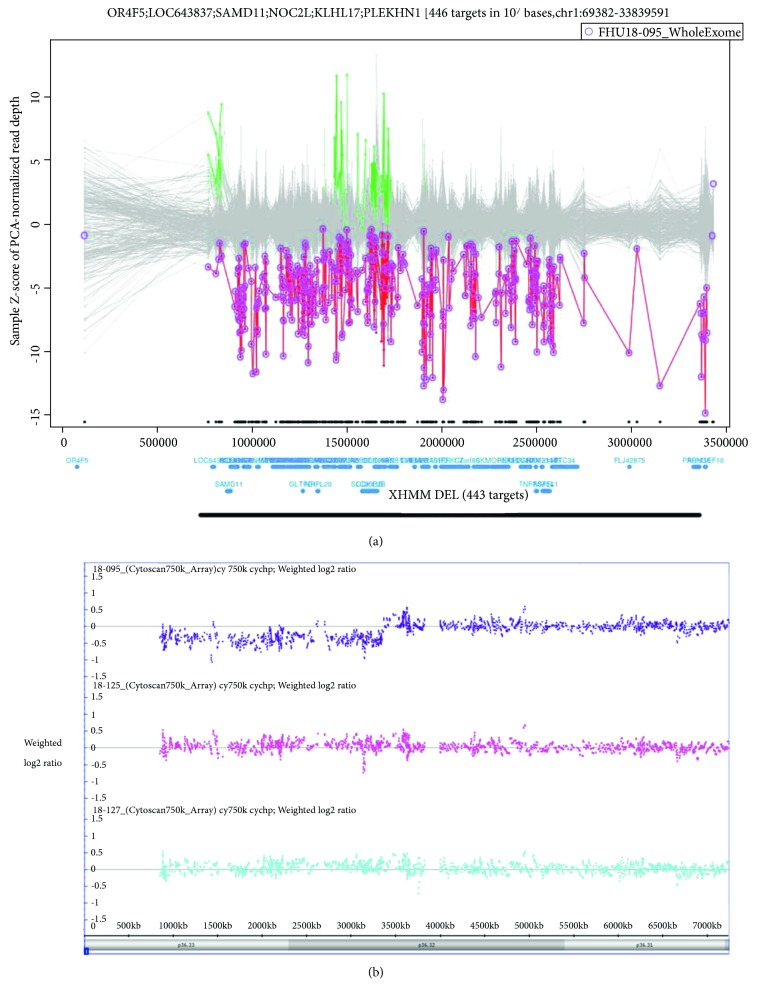
Postnatal molecular cytogenetic analyses. (a) Whole exome sequencing analysis using the eXome Hidden Markov Model (XHMM). XHMM analysis using WES data detected the copy number loss located within 1p36.33–p36.32, suggesting a 3 Mb deletion (black bar). *x* axis shows the physical position, and *y* axis shows the Z score of the principal component analysis that was normalized to read depth. Purple circles connected by red lines represent values of the placenta to WES. Gray dots with gray connected lines indicate the results of normalized read depth. Copy number losses (red dots) without gains (green dots) on chromosome 1p36 were detected in the placenta. (b) Chromosomal microarray (CMA) analysis using Cytoscan 750 k Array. CMA analysis results for the copy number log2 ratio of chromosome 1p region for the placenta (purple), father (pink), and mother (blue). CMA analysis demonstrated a 3 Mb heterozygous deletion within 1p36.33–p36.32 in the placenta. The fetus had arr[hg]1p36.33–p36.32 (849466_3347420)x1. There were no copy number variations in the parents detected by CMA.

**Figure 3:**
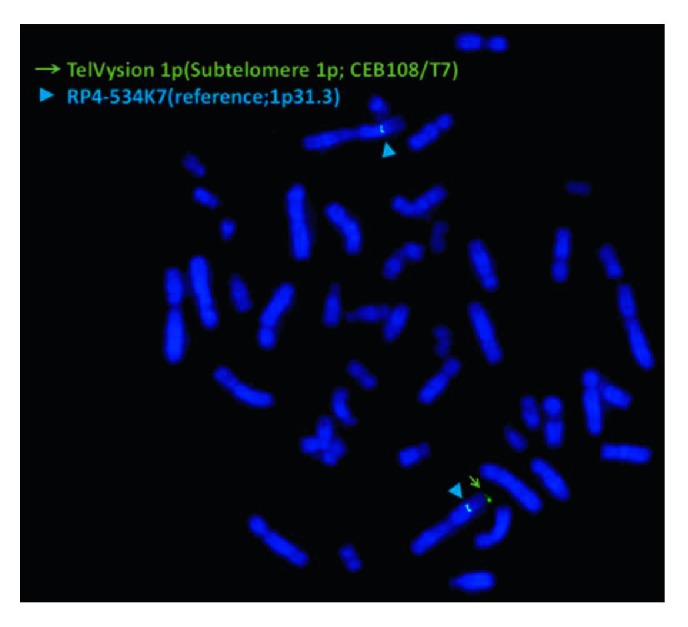
Fluorescence in-situ hybridization (FISH) analysis of cultured amniocytes. FISH showed two 1p31.3 region specific signals (blue) and one 1p36.33 region specific signal (green), indicating 1p36 deletion. The arrow indicates 1p36.33 region specific signal. The arrowhead indicates 1p31.3 region specific signal.

A 28-year-old nulliparous pregnant Japanese woman was referred for prenatal evaluation at 30 weeks of gestation because of abnormal ultrasound findings of fetal congenital heart defects. The family history of the parents was unremarkable. Fetal ultrasonography at 30 weeks and 5 days of gestation showed vascular ring, Ebstein's anomaly, ventricular septal defect, and single umbilical artery. The estimated fetal body weight corresponded to the Japanese standard for the gestational age. Fetal ultrasonography showed vertex presentation of the moving fetus and the fetal stomach appeared to be normally dilated (Table 1). The pregnant woman had not felt any fetal movements since 31 weeks of gestation. At 32 weeks and 5 days of gestation, fetal ultrasonography showed absence of fetal movement with breech presentation, polyhydramnios, absent filling of stomach, and fetal growth restriction (FGR) (Table 1). However, abnormal Doppler findings regarding the fetal middle cerebral artery, umbilical cord artery, and ductus venous were not observed. Clinical diagnosis of fetal akinesia was made at this point. At 32 weeks and 6 days of gestation, amniocentesis was performed to assess the possibility of chromosomal aberrations. Interphase fluorescence in situ hybridization (FISH) analysis on uncultured amniocytes for chromosome 13, 18, and 21 revealed two signals, respectively. At 34 weeks and 6 days of gestation, progression of polyhydramnios with maternal respiratory compromise occurred (Table 1) and 2300 mL of amniotic fluid was removed. G-banding analysis on cultured amniocytes revealed a karyotype of 46,XY,22pstk+ (Figure 1). After discussion with the parents about the prognosis of the fetus based on ultrasound findings, including fetal akinesia since 31 weeks of gestation, FGR, congenital heart defects, and left-sided pleural effusion that indicated severe phenotype with prenatal onset of genetic disorders, perinatal palliative care was chosen. At 36 weeks and 3 days of gestation, fetal ultrasonography showed further progression of polyhydramnios with maternal compromise (Table 1), and 2000 mL of amniotic fluid was removed and labor was induced with oxytocin. The breech neonate was delivered vaginally at 36 weeks and 4 days of gestation with an Apgar score of 1 at 1 min and 1 at 5 min. Birth weight was 1839 g, length 45.5 cm, head circumference 31.8 cm, and chest circumference 23.5 cm. External examination revealed marked muscular hypoplasia of upper and lower extremities, extremely thin transverse palmar creases, joint contractures of lower extremities, hypertelorism, and deep-set ears. The neonate died within 2 h after birth due to respiratory failure. Therefore, we could not assess developmental profile after birth. In addition, permission for neonatal autopsy was not obtained from the parents. Clinical features of the neonate were not sufficient to diagnose a specific disease but suggested the possibility of genetic disorders, including diseases caused by either a single gene or a chromosomal defect. After genetic counseling and obtaining written consent from the parents, whole exome sequencing (WES) was performed with genomic DNA extracted from the placenta using the eXome Hidden Markov Model v1.0 (XHMM). Although the causative gene mutations related to the phenotype of the neonate were not identified, a 3 Mb deletion of chromosome 1p was suspected (Figure 2(a)). The suspected deleted region by the exome analysis using XHMM was further validated by chromosomal microarray (CMA). CMA analysis demonstrated monoallelic deletion located from positions 849466 to 3347420 on chromosome 1p36.33–p36.32 (Chr1:849466–3347420) including 76 genes, which is known as chromosome 1p36 deletion syndrome (Figure 2(b)). Among 76 genes, the gene *SKI*, which is known to contribute to the phenotype of chromosome 1p36 deletion syndrome, is included [4, 7]. These results were consistent with the prenatal sonographic findings and the neonate was diagnosed with chromosome 1p36 deletion syndrome. In addition, CMA analysis revealed no additional copy number variations (CNVs), which suggested *de novo* deletion rather than inheritance from the parents. After genetic counseling for future pregnancies, the parents decided against genetic carrier screening. Postnatal sub-telomeric FISH analysis on cultured amniocytes revealed a terminal deletion of chromosome 1p (Figure 3).

## 3. Discussion

In this report, we present a case of fetal akinesia associated with chromosome 1p36 deletion syndrome diagnosed postnatally by the exome-first approach. To our knowledge, this is the first report describing a case with chromosome 1p36 deletion syndrome presenting with fetal akinesia.

Fetal akinesia is a condition characterized by reduced or absent fetal movement [1–3]. Prenatal sonographic findings of fetal akinesia include lack of extremity motions, persistent abnormal posture of the extremities, polyhydramnios due to decreased fetal swallowing, thorax hypoplasia due to absent fetal breathing, and fetal hydrops [1]. However, these prenatal ultrasound findings are nonspecific to identify the cause, and as yet there is no established approach for prenatal diagnosis of fetal akinesia. Fetal akinesia can result from primary defects at any point along the motor system pathway from the central nervous system to the skeletal muscle cell, which cause diseases such as spinal muscular atrophy, congenital myasthenic syndromes, and congenital muscular dystrophies [1–3]. In addition, a differential diagnosis should include a trisomy 18, metabolic dysfunction such as pyruvate dehydrogenase deficiency, maternal antibodies against acetylcholine receptor, and maternal infections such as cytomegalovirus and toxoplasmosis [1, 8]. A family history is helpful because some diseases are inherited [1–3]. Although a definitive diagnosis helps parents with perinatal decision-making, it would not be possible to do so based on sonographic findings alone. Prenatal sonography could provide sufficient information about a severity of the fetus for parents.

In the present case, while fetal movements and a normal fluid-filled stomach were seen until second trimester, these were absent during the third trimester. In addition, multiple fetal abnormalities such as congenital heart defects, FGR, ventriculomegaly, choroid plexus cysts, and single umbilical artery were found through fetal sonography. Based on these fetal sonographic findings, we performed amniocentesis to rule out trisomy 18. Although the amniocytes showed a normal karyotype, perinatal palliative care was performed based on the prenatal sonographic findings. WES was performed postnatally to assess the possibility of autosomal recessive inherited diseases, including those of neuromuscular origin. As a result, chromosome 1p36 deletion was incidentally identified by quantitative WES analysis using XHMM.

Chromosome 1p36 deletion syndrome causes severe developmental delay, hypotonia, seizure, growth restriction, brain anomalies, and congenital heart defects [4]. Although brain anomalies, FGR, and congenital heart defects in a fetus can be detected using prenatal sonographic examination and indicate the possibility of chromosome 1p36 deletion syndrome [5, 9–11], there is significant phenotypic variation among affected individuals [12]. This phenotypic variation is due, at least in part, to the genetic heterogeneity seen in 1p36 deletions, which include deletions of varying lengths located throughout the 30 Mb of DNA that comprise chromosome 1p36 [6, 12]. In addition, the terminal 1p36 deletion can be missed using conventional G-banding analysis because of the low level of resolution and light staining of the region [5, 11]. In this regard, CMA analysis or subtelomeric FISH may be required to identify chromosome 1p36 deletion [5, 11]. In the present case, a 3 Mb deletion of 1p36 was not seen prenatally in the cultured amniocyte karyotype (Figure 1). Given the WES and CMA findings, the cultured amniocyte karyotype was reanalyzed and the deletion was still not seen. In Japan, the use of sub-telomeric FISH or CMA analysis for prenatal screening are not considered due to legal constraints. However, given the implications for prognosis and higher rate of hypotonia and severe developmental delay, molecular prenatal diagnosis, specifically for deletion of 1p36, should be considered in the setting of a fetal akinesia.

In the present case, the 76 deleted genes included the *SKI* gene that is responsible for the 1p36 deletion phenotype and is one of the candidate genes involved in hypotonia [4, 7]. However, there are no reports of the association between fetal akinesia and 1p36 deletion within a segment from 849466 to 3347420. Four genes *AGRN*, *B3GALT6*, *ATAD3A*, and *PEX10*, which were also among the 76 genes, cause recessive syndrome with hypotonia. As no mutations were detected in the four genes in the nondeleted allele, fetal akinesia may not result from the unmasking of recessive diseases. Therefore, we did not speculate that a deletion located from positions 849466 to 3347420 on chromosome 1p36.33–p36.32 resulted in fetal akinesia, which may present a more severe phenotype with prenatal onset of chromosome 1p36 deletion syndrome. Trio whole genome sequencing is helpful to search other causes of fetal akinesia. Further molecular analyses are essential to clarify this point.

In regard to diagnostic approach for the present case, we performed exome-first approach postnatally following prenatal G-banding analysis. Although the CMA-first approach is still widely used to detect CNVs, WES is becoming available to detect CNVs, leading to an appropriate clinical diagnosis [13]. The advantage of using exome sequencing for a combined analysis of not only single nucleotide variants but also CNVs is to increase the analysis resolution and detection rate with one single test. Therefore, in postnatal testing, WES may be advantageous to screen genetic abnormalities as a first-choice diagnostic approach before performing CMA in undiagnosed syndromic individuals suspected of having either single gene defects or CNVs, such as the present case [13]. After WES analysis, CMA analysis with or without FISH is necessary to accurately determine the range of the region of genomic imbalance for accurate cytogenetic diagnosis.

In conclusion, we propose that chromosome 1p36 deletion syndrome, which may be missed using conventional G-banding karyotype with amniocytes, be considered for differential diagnosis in cases of fetal akinesia and prompt molecular cytogenetic analysis if necessary. As a practical approach to the diagnosis of the cause of fetal akinesia, a detailed anomaly scan should be performed with karyotyping and if unremarkable, exome-first approach may be offered postnatally.

## Figures and Tables

**Table 1:** Prenatal findings of the present case.

GA (Weeks + days)	30 + 5	32 + 5	33 + 3	34 + 6	35 + 5	36 + 3
EFBW (g) (SD)	1353 (−1.2)	1545 (−1.75)	1566 (−2.0)	1694 (−2.27)	1881 (−2.0)	1865 (−2.4)
AFI	9.7	24.4	30.47	48.0	33.2	39.8
Stomach	+	absent	absent	absent	absent	absent
Fetal movements	+	absent	absent	absent	absent	absent
Fetal presentation	Vertex	Breech	Breech	Breech	Breech	Breech
Ventriculomegaly (mm)	4.4	8.2	10	14.2	15	N/A
Others		CPCs		PE	PE	PE

GA, gestational age; EFBW, estimated fetal body weight; AFI, amniotic fluid index; CPCs, choroid plexus cysts; PE, pleural effusion; SD, standard deviation; N/A, not available.

## References

[1] Hellmund A., Berg C., Geipel A., Müller A., Gembruch U. (2016). Prenatal diagnosis of fetal akinesia deformation sequence (FADS): a study of 79 consecutive cases. *Archives of Gynecology and Obstetrics*.

[2] Ravenscroft G., Sollis E., Charles A. K., North K. N., Baynam G., Laing N. G. (2011). Fetal akinesia: review of the genetics of the neuromuscular causes. *Journal of Medical Genetics*.

[3] Beecroft S. J., Lombard M., Mowat D. (2018). Genetics of neuromuscular fetal akinesia in the genomics era. *Journal of Medical Genetics*.

[4] Jordan V. K., Zaveri H. P., Scott D. A. (2015). 1p36 deletion syndrome: an update. *The Application of Clinical Genetics*.

[5] Campeau P. M., Ah Mew N., Cartier L. (2008). Prenatal diagnosis of monosomy 1p36: a focus on brain abnormalities and a review of the literature. *American Journal of Medical Genetics Part A*.

[6] Gajecka M., Mackay K. L., Shaffer L. G. (2007). Monosomy 1p36 deletion syndrome. *American Journal of Medical Genetics Part C: Seminars in Medical Genetics*.

[7] Okamoto N., Toribe Y., Nakajima T. (2002). A girl with 1p36 deletion syndrome and congenital fiber type disproportion myopathy. *Journal of Human Genetics*.

[8] Winters L., Van Hoof E., De Catte L. (2017). Massive parallel sequencing identifies RAPSN and PDHA1 mutations causing fetal akinesia deformation sequence. *European Journal of Paediatric Neurology*.

[9] Seo G. H., Kim J. H., Cho J. H. (2016). Identification of 1p36 deletion syndrome in patients with facial dysmorphism and developmental delay. *Korean Journal of Pediatrics*.

[10] Chen C. P., Chen M., Su Y. N. (2010). Chromosome 1p36 deletion syndrome: prenatal diagnosis, molecular cytogenetic characterization and fetal ultrasound findings. *Taiwanese Journal of Obstetrics and Gynecology*.

[11] Lissauer D., Larkins S. A., Sharif S., MacPherson L., Rhodes C., Kilby M. D. (2007). Prenatal diagnosis and prenatal imaging features of fetal monosomy 1p36. *Prenatal Diagnosis*.

[12] Rocha C. F., Vasques R. B., Santos S. R., Paiva C. L. (2016). Mini-Review: Monosomy 1p36 syndrome: reviewing the correlation between deletion sizes and phenotypes. *Genetics and Molecular Research*.

[13] Watanabe M., Hayabuchi Y., Ono A. (2016). Detection of 1p36 deletion by clinical exome-first diagnostic approach. *Human Genome Variation*.

